# Amplified Spontaneous Emission Properties of Semiconducting Organic Materials

**DOI:** 10.3390/ijms11062546

**Published:** 2010-06-18

**Authors:** Eva M. Calzado, Pedro G. Boj, María A. Díaz-García

**Affiliations:** 1 Departamento Física, Ingeniería de Sistemas y Teoría de la Señal and Instituto Universitario de Materiales de Alicante, Universidad de Alicante, Alicante-03080, Spain; E-Mail: evace@ua.es; 2 Departamento Óptica and Instituto Universitario de Materiales de Alicante, Universidad de Alicante, Alicante-03080, Spain; E-Mail: p.boj@ua.es; 3 Departamento Física Aplicada, Unidad asociada UA-CSIC and Instituto Universitario de Materiales de Alicante, Universidad de Alicante, Alicante-03080, Spain

**Keywords:** lasers, semiconducting organic materials, oligomers, amplified spontaneous emission

## Abstract

This paper aims to review the recent advances achieved in the field of organic solid-state lasers with respect to the usage of semiconducting organic molecules and oligomers in the form of thin films as active laser media. We mainly focus on the work performed in the last few years by our research group. The amplified spontaneous emission (ASE) properties, by optical pump, of various types of molecules doped into polystyrene films in waveguide configuration, are described. The various systems investigated include *N*,*N*′-bis(3-methylphenyl)-*N*,*N*′-diphenylbenzidine (TPD), several perilenediimide derivatives (PDIs), as well as two oligo-phenylenevinylene derivatives. The ASE characteristics, *i.e.*, threshold, emission wavelength, linewidth, and photostability are compared with that of other molecular materials investigated in the literature.

## 1. Introduction

In recent years, great effort has been devoted to the development of organic solid-state lasers [1,2]. A very unique property of organic materials is that, due to their broad photoluminescence (PL) spectrum, a laser wavelength produced from them can be turned over a wide range [3]. Among the various types of organic materials, those that are soluble have the advantage of easy processability. They can be deposited in the form of thin films by inexpensive techniques. Organic solid-state lasers consisting of dyes incorporated into solid matrices were demonstrated in the seventies [4]. However, these materials have serious problems of photostability. They cannot be pumped electrically and generally, there is a limit in the concentration of dye that can be introduced into the matrix, since molecular interactions lead to PL quenching [3]. As a consequence, the development of commercial organic solid-state lasers has not been viable. In 1996, interest in the field was renewed with the discovery of stimulated emission in semiconducting polymer films [5–7]. Since then, many semiconducting materials, including small molecules, oligomers, dendrimers, and polymers, have been investigated in devices with different types of configurations, such as microcavities, distributed feedback lasers, *etc.* [1,2]. These materials could potentially solve some of the aforementioned problems with respect to the dyes. Firstly, these materials are semiconductors; they open the possibility of electrical pumping. Secondly, many of these semiconducting materials did not show limitations in the concentration of active material. Thus, stimulated emission could be obtained from neat (non-diluted) films, leading to much lower laser thresholds. Photostability has constituted the main focus of the work done in the last years within the field of traditional dyes. But, there is little information about the photostability of most of the semiconducting materials studied. This issue remains one of the most important challenges for the realization of commercial systems. Nowadays, a great advance on optically pumped structures has occurred [2]. For some materials, thresholds are low enough to be pumped with microlasers that are the size of a match box [8], and even with GaN lasers [9]. As a result, applications based on optically pumped structures are becoming a reality.

The easiest way to evaluate the potential of a certain material to be used as an active laser medium, as well as to compare its performance with that of other materials, consists of photopumping films of the material and identifying the collapse of width in their PL spectra at a certain pump intensity [1,6]. This spectral collapse is normally accompanied by a large enhancement of the output intensity and accounts for the presence of gain due to stimulated emission. Although these structures provide light amplification, they are not real lasers. No laser modes are present and these structures possess a limited degree of coherence and monochromaticity. Nevertheless, this is the most appropriate technique for properly comparing the performance of different materials. The technique allows the identification of the variations in their behavior that are due to the material, rather than the effect of the resonant cavity. Moreover, these studies allow one to establish structure-property relationships that provide useful hints to organic chemists for the improvement of materials design for laser applications.

There exist different possible mechanisms that can cause this spectral collapse [10]. When the active films constitute waveguides (*i.e.,* the refractive index of the film is larger than that of the substrate, and the film thickness is sufficient to support modes), the spectral narrowing generally results from amplification of spontaneous emission (ASE) due to stimulated emission. However, reports on other materials have attributed spectral narrowing to other mechanisms, such as superfluorescence or interacting excitons. A way to identify the mechanism responsible for the spectral collapse consists of investigating the dependence of the emitted intensity on the length of the pump stripe [11].

If ASE occurs, the spectra should be broad at short stripe lengths and should narrow as the excitation length increases. Moreover, the output intensity at the end of the stripe should follow the expression:

(1) $$I(\lambda )=\left\lfloor A(\lambda )I_{p}/g(\lambda )\right\rfloor \{\text{exp}[g(\lambda )l]-1\}$$

where *A(*λ*)* is a constant related to the cross section for spontaneous emission, *I*_p_ is the pump intensity, *g(*λ*)* is the net gain coefficient, and *l* is the length of the pump stripe. In contrast, if superfluorescence or biexcitonic emission is the mechanism of spectral narrowing, the width of the emission spectrum should not depend on the size of the excited region, and the output intensity should only increase linearly with the length of the excited region (or sublinearly if the waveguide losses are substantial) [10,11].

**Figure 1.**
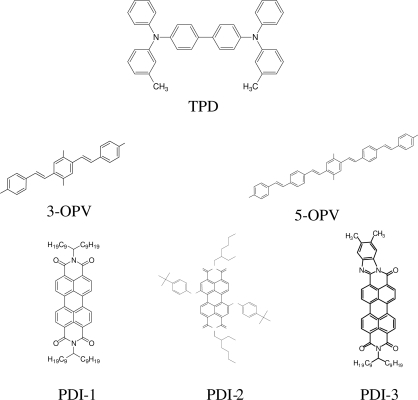
Chemical structure of compounds investigated in this work.

Among the various classes of semiconducting materials, our research group has focussed on small organic molecules and oligomers. In the last years, the laser properties of many systems of this class have been investigated [1,2]. Their ASE, or laser thresholds, are often much lower than those of traditional dyes [12], and comparable to those of various semiconducting polymers [1,2]. This is because in many cases, semiconducting molecules can be doped into the films at larger concentrations, without getting PL quenching. Note that the typical concentrations used with dyes are around 1 wt %. Moreover, for some particular molecular semiconducting materials, ASE was observed even in the form of neat films (non-diluted in an inert matrix) [13–15]. An example of such materials is the hole-transporting N,N′-bis(3-methylphenyl)-N,N′-diphenylbenzidine system (TPD) [16–19] (Figure 1). Since film quality and supramolecular organization play a major role in obtaining high PL efficiencies and stimulated emission in the solid state, in the last few years, our group has insisted upon the importance of performing detailed investigations of ASE performance as a function of the concentration of active molecule in the film. Thus, such studies have been carried out with TPD [19], with two oligo-(*p*-phenylenevinylene) derivatives (OPV-3 and OPV-5) [20], and with three perylenediimide derivatives (PDI-1, PDI-2 and PDI-3) (see chemical structures in Figure 1) [21,22]. The interest in using these materials is discussed below.

The potential of PDIs in solution for laser applications was first demonstrated by Sadrai *et al.* [23]. Most subsequent works dealt with the commercially available perylene orange and perylene red, either in solution [24,25] or incorporated in solid matrices [26–32]. In addition, most of the work performed in solid state has focused on using sol-gel matrices, with the aim of improving photostability. PDIs are among the most photostable materials reported in the literature. This is their most attractive property for laser applications. However, no detailed investigations of the dependence of laser performance on PDI concentration had been done. With respect to such a study, the biggest challenge would be to achieve concentrations of PDI that are as large as possible, so as to produce lower laser thresholds, while maintaining a good photostability. Another aspect of interest of PDIs is that they emit at wavelengths that can easily match the low-transmission windows in poly(methyl methacrylate) (PMMA) at 530, 570, and 650 nm. Thus, PDIs have a great potential in the field of data communications, where polymer optical fibers are used, such as fibers used in the home or workplace and in data transfer within automobiles [2]. Within this context, we investigated the ASE properties of various PDI derivatives (PDI-1, PDI-2 and PDI-33) doped into polystyrene (PS) films. The aim was to obtain low laser thresholds and obtain high photostabilities, as well as to develop the capability of tuning the emission wavelength to better match the PMMA windows. It is well known that the absorption spectra of PDIs symmetrically substituted at the imide nitrogen positions, such as PDI-11, are strongly structured and are only slightly influenced by solvent effects and by the type of substituents at the N positions [33–35]. Consequently, their absorption spectra cannot be tuned by substitution at the N atoms. These types of derivatives are planar, very photostable, and also very stable thermally. In addition, they show very high PL quantum yields in solution (Φ □ ≈ □1) [34,36] and, for some derivatives (such as PDI-1), high yields were also observed in the solid state [34]. There are mainly two routes [34] for tuning the absorption spectra of PDIs: substitution at the perylene core and modification of the dicarboximide group. The most interesting aspect of PDIs substituted at the bay positions of the core (such as PDI-2), is that the π-□π stacking energy is significantly reduced by the steric hindrance induced by the bay substituents. The stacking energy is minimal for the most twisted π-systems [38]. Since intermolecular interaction generally leads to a decrease in the PL efficiency, and consequently, in the laser performance, one can expect to achieve laser emission with larger concentrations of the active material in the film, as compared to PDIs substituted at the imide nitrogen positions. Nevertheless, it should be noted that substitution at the bay positions of the perylene core generally leads to a decrease of the PL quantum yield that would be negative for the laser’s performance. For example, Φ = 0.85 and Φ = 0.5 have been measured in a tetracloro-substituted perylene [25] and in a cholesterol-based perylene, respectively [39]. On the other hand, the route consisting of modifying the dicarboximide group in the PDI (PDI-3), leads to more drastic changes in the absorption spectrum of the monomer, while the π- □π stacking energy remains practically unchanged.

Concerning OPV derivatives, Kretsch *et al.* reported ASE emission [40], as well as DFB laser action [41], in polystyrene films doped with 1,4-bis-(b-cyanostyryl)-2,5-dimethoxybenzene. It should be noted that most of the early work done in the field of polymer lasers involves Poly-(*p-*phenylenevinylene) (PPV) derivatives. The interest in using oligomers resides in the fact that in contrast to polymers, a perfect control of the length of the chain is achieved [42,43]. Thus, these systems serve as models for investigating the influence of inter- and intra-chain interactions in the stimulated emission processes. In our research group, two different OPV derivatives (with three and five monomeric units) were investigated. We were particularly interested in two issues. First, by comparing both compounds, we could study the influence of the length of chain on emission processes. Second, by changing the concentration of the oligomer in the films, the role of possible aggregation mechanisms, as well as interchain interactions, could be explored.

In this review, we compile the results achieved in the last years by our research group in the field of organic solid-state lasers. The capability of polymeric films doped with different molecules and oligomers to show ASE and thus, to act as active laser materials, has been investigated. The various molecular systems under study include (see Figure 1): TPD, two different OPV′s, one with three (3-OPV) and the other with five (5-OPV) monomeric units, and three PDI derivatives. The three are PDI-1, which is symmetrically substituted at the imide nitrogen positions, PDI-2, which is substituted at the bay position in the PDI core, and PDI-3, which is modified in the dicarboximide group.

## 2. Results and Discussion

### 2.1. Identification of Stimulated Emission and Definition of Threshold

**Figure 2.**
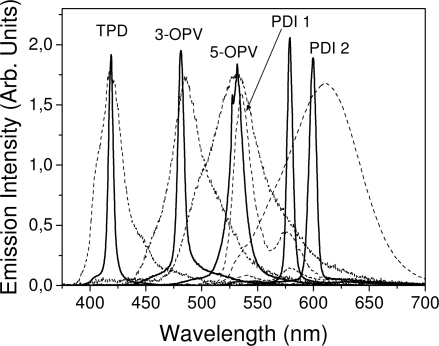
Emission spectra at low and high pump intensities for PS films doped with optimal concentrations (C_o_) of TPD (30 wt %), 3-OPV (15 wt %), 5-OPV (9 wt %), PDI-1 (0.75 wt %), and PDI-2 (2.8 wt %).

Emission spectra of PS films doped, at various concentrations, with the different materials shown in Figure 1, were obtained in the setup for the identification of stimulated emission (see the experimental section). Films were excited at a wavelength close to their maximum absorption: 355 nm for those doped with oligomers and with TPD, and 533 nm for those based on PDI derivatives. Figure 2 shows spectra obtained at low and high pump intensities for the various materials. The doping concentration in each case corresponds to the so-called optimal concentration (C_o_), *i.e.*, that at which the lowest thresholds were obtained. A detailed discussion about the concentration dependence will be given in section 2.3. In all cases, except for that of PDI-3, a collapse of the emission spectrum at a certain pump intensity was observed. The existence of spectral narrowing is a signature of the presence of stimulated emission. It is observed that these derivatives cover a wide range of emission wavelengths: 417 nm (TPD), 480 nm (3-OPV), 530 nm (5-OPV), 581 nm (PDI-1) and 599 nm (PDI-2).

**Figure 3.**
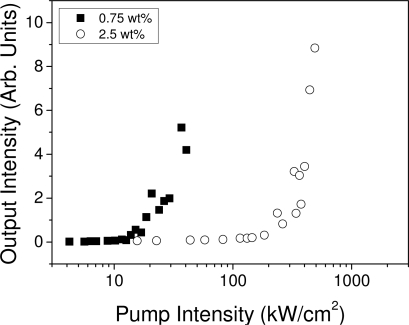
Output intensity at λ = 579 nm as a function of pump intensity for PS films doped with 0.75 and 2.5 wt % of PDI-1.

**Figure 4.**
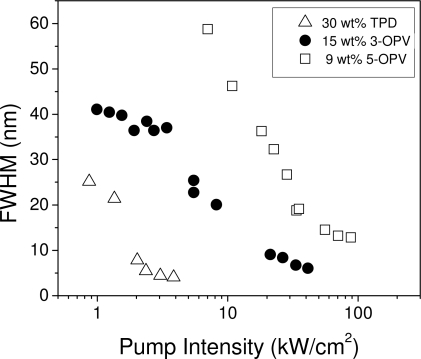
Full width at half of the maximum (FWHM) as a function of pump intensity for PS films doped with 3-OPV, 5-OPV, and TPD at various concentrations.

As a result of the existence of gain, besides the narrowing of the emission spectrum at a given pump intensity, a considerable increase in the output intensity was observed. Results obtained for PDI-1-doped films are shown in Figure 3, where the output intensity at the wavelength of the peak emission has been represented as a function of pump intensity. As observed, the intensity grows with the pump intensity and shows a clear change in slope at a certain pump intensity. The pump intensity, at which both a spectral collapse and a drastic change in the output intensity are observed, is called the “threshold”. This threshold is generally determined from the curves representing the emission linewidth (full width at half of the maximum, FWHM) as a function of pump intensity. The threshold is the intensity at which the FWHM decays to half of its maximum value. This method for the determination of the threshold is illustrated in Figure 4 for various materials. The threshold can also be determined as the intensity at which a change of slope is observed within an output *versus* input intensity curve (see Figure 3). For PDIs, this method is more appropriate; since PL spectra show two peaks that make a proper determination of the FWHM difficult. It should also be noted that thresholds determined from the change of slope are slightly higher than those obtained from the FWHM decay because the change of slope takes place when the spectrum is already narrow.

### 2.2. Determination of the Mechanism Responsible for the Observation of Gain-Narrowing

**Figure 5.**
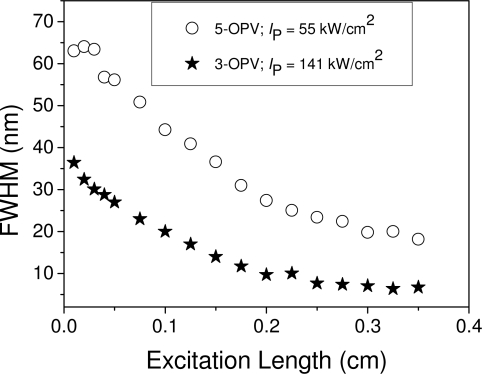
FWHM as a function of excitation length, for PS films doped with 15 wt % of 3-OPV and 9 wt % of 5-OPV.

**Figure 6.**
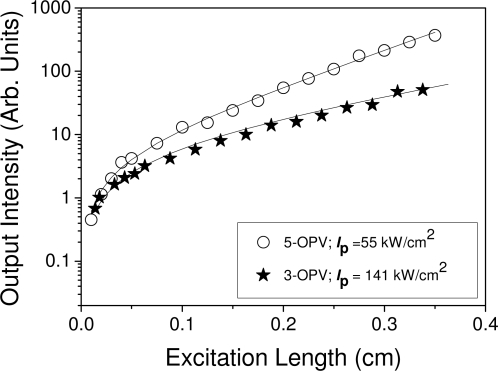
Emission intensity at λ = 480 nm (for 3-OPV) and λ = 530 nm (for 5-OPV) as a function of excitation length for PS films doped with 15 wt % of 3-OPV and 9 wt % of 5-OPV. The solid lines are the fit to data using Equation (1).

We studied the dependence of the emission spectrum on the length of the pump stripe in order to identify the mechanism responsible for the observation of gain. Figures 5 and 6 show the results obtained for the OPV derivatives. As shown in Figure 5, the spectra are broad for short pump stripes and become narrower when the pump stripe gets larger. This indicates that the responsible mechanism is ASE. In addition, the dependence of the output intensity at the peak of the emission spectrum (λ = 480 nm and λ = 530 nm for 3-OPV and 5-OPV, respectively) on the pump stripe length (Figure 6) can be adequately fitted to Equation (1), thus providing additional support for the assignment of ASE as the mechanism accounting for the observation of gain in these systems. The same type of behavior was observed for the other derivatives investigated. The parameter, A*I*_p_, and the net gain coefficients, *g,* obtained from the fits of the corresponding curves of the various materials investigated in this work are shown in Table 1. It is observed that for a certain material, gain coefficients get larger when the pump intensity increases.

### 2.3. Concentration Dependence of ASE

The dependence of ASE on the concentration of active material in the films was ascertained for the various molecular systems investigated in this work. The range of concentrations over which ASE was observed was rather different for the various materials (see Table 2). TPD shows the widest range, since ASE was observed for all concentrations between 2.5 and 100 wt %. For the OPV derivatives, this range is more limited. For films doped with 3-OPV, ASE was observed up to concentrations of around 25 wt %. For larger concentrations, spectral narrowing was still observed, but at such large pump intensities, films degraded very quickly [20]. The limiting concentration for films doped with 5-OPV was also 25 wt %, although in this case no ASE was observed at higher concentrations. Concerning PDIs, the range of concentrations for the observation of ASE is even more restricted. For films doped with PDI-1 and PDI-2, ASE was observed for concentrations between 0.25 and 5 wt % and between 1.5 and 3 wt %, respectively. On the other hand, no ASE was observed in films doped with PDI-3, no matter how strong the pump intensity was. This is probably due to its low PL quantum yield (φ ≈ 0.2 ± 0.1).

In what follows, we describe the effect of changing the concentration of the active molecule on the various ASE parameters: the pump intensity threshold (*I*_th-ASE_), the linewidth above threshold (FWHM_ASE_), the emission wavelength (λ_ASE_), and the photostability.

#### 2.3.1. ASE Thresholds

ASE thresholds were determined for films doped with various materials at different concentrations, following the methods described in section 2.1. In the case of TPD and OPVs, *I*_th-ASE_ values were calculated as the intensity at which the FWHM of the emission spectrum decreases at half of its maximum value (see Figure 5). On the other hand, as already discussed, for PDIs, thresholds were obtained from the output *versus* input intensity curves (Figure 4).

For all the materials investigated, *I*_th-ASE_ decreased when the concentration was increased up to a certain value. These values differed with the material: 20 wt % for TPD, 15 wt % for 3-OPV, 9 wt % for 5-OPV, and 0.75 wt % for PDI-1. The behavior above this concentration is also different for the various systems. For TPD, the threshold remains approximately constant up to very high concentrations. For the other materials, the ASE threshold increases. The initial decrease of the threshold at low concentrations is explained by the fact that the number of active molecules in the illuminated area increases. The reason for the existence of a limiting concentration, above which the ASE threshold saturates or increases, is due to the existence of some kind of aggregation or interaction mechanism, or both, that leads to PL and ASE quenching. In some cases, the presence of aggregated species was evidenced by the observation of changes in the absorption spectra (5-OPV) [20] or in the PL spectra (PDIs) [22]. On the other hand, in other materials, such as TPD, both absorption and PL spectra remain unchanged with concentration. Recent calculations based on density functional theory, together with resonant Raman spectroscopy, have been used to propose a model to explain this behavior [44]. Results have shown that the influence of molecular interactions is much smaller than that of the torsional modes of TPD at very low frequency. Thus, TPD’s photophysics can be understood from the properties of the molecule itself, even for highly-doped films.

The concentration at which *I*_th-ASE_ either saturates or either starts increasing, is the one at which *I*_th-ASE_ has the lowest value. These concentrations, called optimal concentrations (C_o_), together with their corresponding *I*_th-ASE_ values for the various materials under investigation, are listed in Table 2. As observed, TPD shows the best performance. TPD is the material that can be doped at a higher concentration without getting ASE quenching. Concerning the OPVs, apparently the thresholds obtained for 3-OPV and 5-OPV are similar. However, taking into account that their molecular weight is different, it can be concluded that 5-OPV is more efficient than 3-OPV [20]. Finally, with respect to the PDIs, clearly PDI-1 is the best-performing one, showing a threshold of 15 kW/cm^2^ when doped at a concentration as low as 0.75 wt %. A much larger threshold was measured for PDI-2 and no ASE was observed for PDI-3. The reason for such low limiting concentrations is the appearance of aggregated species. Precisely for concentrations above the optimal concentration, an additional peak appears at around 600 nm that is related to the presence of excimers [21].

#### 2.3.2. ASE Linewidths

The ASE linewidth (FWHM_ASE_ measured at a pump intensity above the threshold) also varies as a function of the type of active molecule in the film. For all the materials investigated, the FWHM_ASE_ decreases with increasing concentration up to a certain concentration, generally coincident with the one at which the minimum threshold was measured. Above this value, the FWHM_ASE_ saturates. The FWHM_ASE_ values measured at the optimal concentration are listed in Table 2.

As observed, the lowest linewidths (around 5 nm), have been obtained for TPD. Concerning the OPVs, films doped with 3-OPV showed a better performance, with a linewidth of 6 nm, in contrast with the 12 nm obtained for 5-OPV.

#### 2.3.3. ASE Wavelengths

**Figure 7.**
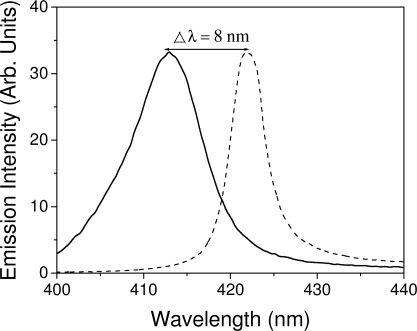
ASE spectra for PS films doped with 2.5 wt % (solid line) and 70 wt % (dashed line) of TPD.

Among the various materials studied here, the only one that has shown significant changes in the emission wavelength when changing the concentration has been TPD. In fact, this is expected due to its much larger range of operation. In this case, λ_ASE_ can be tuned between 413 and 421 nm by changing the concentration of TPD from 2.5 to 70 wt % (See Figure 7). When the concentration of TPD increases, the absorption at shorter wavelengths also increases. Since at these wavelengths absorption overlaps with the PL, the emission is forced to shift to longer wavelengths. It should be noted that the shifts observed in the ASE were also observed in the PL emission.

As already mentioned previously, for the OPVs, ASE takes place at λ_ASE_ = 480 nm and at λ_ASE_ = ≈530 nm for films doped with 3-OPV and 5-OPV respectively. These values remain approximately constant for all the concentrations. The same behavior was observed for films doped with PDI-2, for which λ_ASE_ = 599 nm in the range of concentrations studied and no displacement of the ASE wavelength was observed. For PDI-1-doped films, the ASE wavelength changes very slightly from low concentrations (λ_ASE_ = 579 nm) to higher concentrations (λ_ASE_ = 581 nm).

#### 2.3.4. Photostability

Generally, photostability is evaluated by recording the total ASE intensity emitted under a constant pump intensity just above the threshold, as a function of time. The presence of photodegradation is identified when a decrease of the total ASE output is observed. The parameter employed to characterize such property is the so-called photostability half-life (*t*_1/2-ASE_), defined as the time (or the number of pump pulses) at which the ASE intensity decays to half of its maximum value.

**Figure 8.**
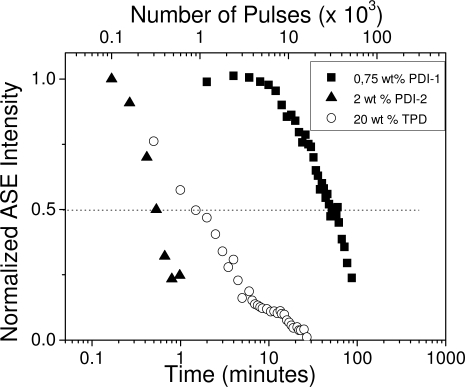
Normalized ASE intensity *versus* irradiation time (bottom axis) and *versus* the number of pulses (top axis) for PS films doped with different TPD, PDI 1 and PDI 2 concentrations.

Figure 8 shows the results obtained for films doped with representative concentrations of various materials: PDI-1 (0.75 wt %), PDI-2 (2 wt %) and TPD (20 wt %). The pump intensities were around 70, 2500 and 5 kW/cm^2^ respectively. As observed, the best results were obtained for PDI-1, that shows an ASE lifetime of 31 × 10^3^ pulses (*i.e.*, 50 min) [21,22]. On the other hand, the film doped with PDI-2 degraded very quickly due to its larger ASE threshold, showing a *t*_1/2-ASE_ of only 300 pulses [18]. In the case of TPD, the ASE output decreases to half of its maximum value at around 1500–1750 pulses (2.5–3 minutes) [19]. The *t*_1/2-ASE_ values measured at the optimal concentrations for all the materials, including the OPVs, are listed in Table 2.

The dependence of the photostability on the concentration was also investigated. It was observed that photostability decreased with increasing concentration. This was due in part to the higher pump intensities needed to obtain ASE.

### 2.4. Comparison of the ASE Performance of Various Materials

Finally, in order to highlight the advantages and limitations of the materials studied here, their performance is compared to that of various representative materials studied in the literature, both molecular and polymeric (see Table 3 (a) and 3 (b)). The two parameters used for this comparison are the ASE threshold and the photostability half-life. Concerning the ASE threshold, in order to compare different materials, it is important to give this parameter in power density units (W/cm^2^) [21]. In addition, ASE thresholds, rather than laser thresholds (with cavities), should be considered. Otherwise, it would not be clear whether the improvement in performance is due to the material or to the cavity. It is important to note that if materials are not pumped close to the peak of the absorption band, some kind of normalization should be done in order to compare the performance. In our case, we normally pumped close to the absorption peak, so normalization is not necessary. First of all, among the various materials studied in this work, the best-performing one, from the point of view of photostability, has been PDI-1 (see Table 2). On the other hand, in terms of ASE threshold, the best results were obtained with TPD.

By comparing Tables 2 and 3, it is clear that PDI-1-based films are among the most photostable materials studied. Moreover, the films’ photostability could probably be improved further by the usage of other matrices that are different from that of PS. It should also be noted that there is practically no information in the literature about the photostability of polymers used for laser applications. In addition, PDI-1-based materials show ASE thresholds lower than those obtained with films based on other molecular materials, such as PM567 [12] or T5oxPh [49]. This result is particularly remarkable, given that the amount of PDI in the films is only 0.75 wt %, in contrast with the larger concentrations used with T5oxPh. This fact is also important when comparing PDI 1 with polymers. Although the ASE threshold for PDI 1-doped films is one order of magnitude larger than those of some of the polymers shown in the table, this difference is not so significant if one considers that there are two orders of magnitude difference in the concentration [21].

Concerning TPD, it presents an ASE threshold [19] as low as 1 kW/cm^2^ (when doped at 30 wt % into PS), that is among the lowest reported in the literature for molecular materials. Moreover, this is comparable to those of typical semiconducting polymers emitting in similar regions of the optical spectrum (see Tables 2 and 3).

Finally, the ASE properties of films doped with 3-OPV and 5-OPV are comparable to those of films based on others oligomers, such as FTS, T3oxPh, and T5oxPh [48,49], where the concentration of active material is higher.

## 3. Experimental Section

Films of an inert polymer (polystyrene PS), doped with TPD, an oligomer (3-OPV, 5-OPV) or a PDI derivative (PDI-1, PDI-2, PDI-3), were deposited over glass substrates by the spin-coating technique. The solvent was toluene in all cases, except for PDI-3, where chloroform was used. The films’ quality transparency and homogeneity were very good and their stability and performance lasted for many months. The film thickness, measured by means of an interferometer coupled to an optical microscope, was approximately 425 nm for TPD films, 1 μm for PDI derivative films, and varied between 320 nm and 580 nm for 3-OPV, and from 400 nm to 620 nm for 5-OPV. In all cases, film thickness was well above the cut-off thickness for the propagation of one mode. Thus mode confinement was not influencing the ASE thresholds [18].

Absorption and PL spectra were obtained using a Shimadzu spectrophotometer and a Jasco FP-6500/6600 fluorimeter, respectively. In the fluorimeter, samples were excited at 355 nm (TPD and oligomers), 491 nm (PDI compound 1), and 533 nm (PDI compounds 2 and 3). The beam was collected at a 45º angle to avoid the pump beam.

**Figure 9.**
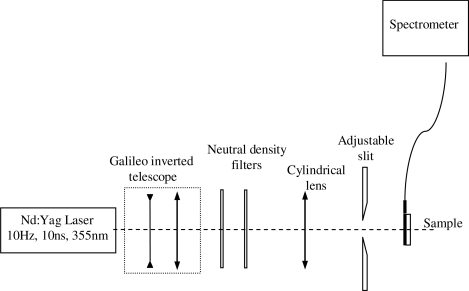
Experimental setup for characterizing the presence of stimulated emission in waveguide configuration.

The experimental setup used to investigate the presence of stimulated emission in waveguide configuration is shown in Figure 9 [11]. Samples were photopumped at normal incidence with a pulsed Nd:YAG laser (10 ns, 10 Hz) operating at 355 nm, which lies in the absorption region of TPD and oligomers, and 532 nm for PDI derivatives. The energy of the pulses was controlled using neutral density filters. The laser beam was expanded and collimated. A cylindrical lens and an adjustable slit were then used to shape the central part of the beam into a stripe with a width of approximately 0.53 mm and a length that could be varied between 0.1 and 3.5 mm. The pump stripe was projected right up to the edge of the film, where the emitted light was collected with a fiber spectrometer.

The photostability experiments were performed in ambient conditions, and not in vacuum, because ideally, the devices should work in ambient conditions. If experiments were conducted in a vacuum, the photostability would improve, but then, the material would be more limited from the point of view of applications.

## 4. Conclusions

We have reported the amplified spontaneous emission properties of polymer films doped with various types of organic semiconducting molecules: *N*,*N*′-bis(3-methylphenyl)-*N*,*N*′-diphenylbenzidine (TPD), two different oligo-(*p*-phenylenevinylene) derivatives (OPVs), with three (3-OPV) and five (5-OPV) monomeric units, and three perylenediimide derivatives (PDIs). Detailed studies of the dependence of their amplified spontaneous emission properties on the concentration of active material have allowed one to optimize their performance. The best results were obtained for TPD and for the PDI derivative symmetrically substituted at the imide positions (PDI-1). TPD shows stimulated emission at any concentration, even in the form of neat films. It shows an ASE threshold as low as 1 kW/cm^2^ when doped at concentrations of 20 wt % or higher. This threshold is among the lowest for molecular materials. Concerning films based on PDI-1, they present a very good photostability half-life (31000 pulses) and a reasonable ASE threshold (15 kW/cm^2^) when doped at a concentration of only 0.75 wt %.

## Figures and Tables

**Table 1.** Parameters obtained from fits to Equation (1) for PS films doped with various materials at the indicated concentrations.

**Material (concentration)**	**Pump Intensity (kW/cm^2^)**	***AI_p_* (arb. units)**	***g* (cm^−1^)**
3-OPV (15 wt %)	100.0	22.6	4.3
	141.0	42.3	6.5
5-OPV (9 wt %)	27.0	29.6	4.0
	34.0	39.7	9.0
	55.0	57.2	13.0
TPD (10 wt %)	6.2	58.2	0.5
	10.5	16.7	14.0
	12.4	22.5	16.0
PDI–1 (0.5 wt %)	26.0	2.7	2.0
	60.0	7.9	8.3
	74.0	9.6	10.0

**Table 2.** Comparison of the ASE performance of PS films doped with various materials.

**Material^a^**	**C_r_^b^**	**C_o_^c^**	λ_ASE_^d^*	FWHM_ASE_^e^*	*I*_th-ASE_^f^*	*t*_1/2-ASE_^g^*	**Ref^h^**
TPD	2.5–100	30	417	5	1	1500	19
3-OPV	2–30	15	480	6	6	600	20
5-OPV	2–25	9	530	12	13	2000	20
PDI-1	0.25–5	0.75	579	7	15	31000	22

a Material: Active material;

b C_r_: Concentration range of active material (wt %) over which ASE is observed;

c C_o_: Optimal concentration of active material (wt %);

d □_ASE_: ASE wavelength (nm);

e FWHM_ASE_: ASE linewidth (nm);

f *I*_th-ASE_: Pump intensity thresholds for ASE observation (kW/cm^2^);

g *t*_(1/2)-ASE_ : Photostability half-life (pump pulses);

h Ref: Reference;

* Parameters measured in films doped at optimal concentrations.

**Table 3.** (a) Molecular materials; (b) Polymeric materials.

**Material (3.a)**	**Structure^a^**	***λ*ASE^b^**	***I*th-ASE^c^**	***t*1/2-ASE^d^**	**Type of sample^e^**	**Ref^f^**
Spiro-4p	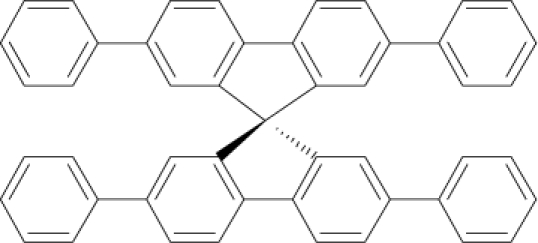					
Neat film		390	31	---	SC	45
Spiro-6p	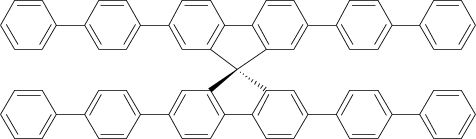					
Neat film		425	3	1 × 10^4^	SC	45
BTAPVB/PS	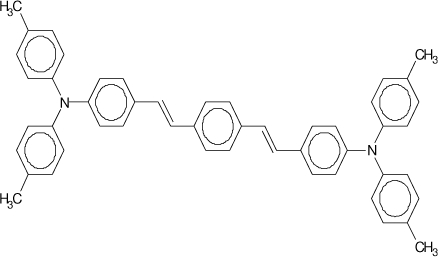					
9:1		504	300	---	SC	46
IR140/PMMA	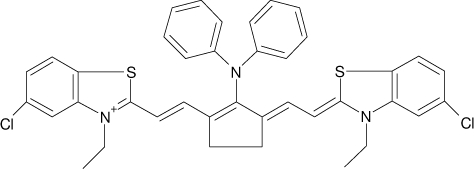					
0.0015:1		970	1600	---	SC	47
PM567/PMMA	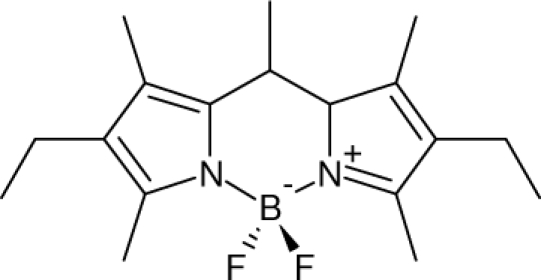	569	3800	16 × 10^3^		12
FTS	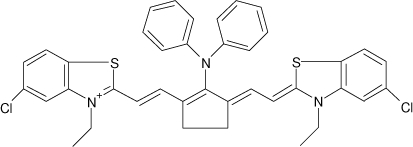	542	50	---	SC	48
T3oxPh/PC	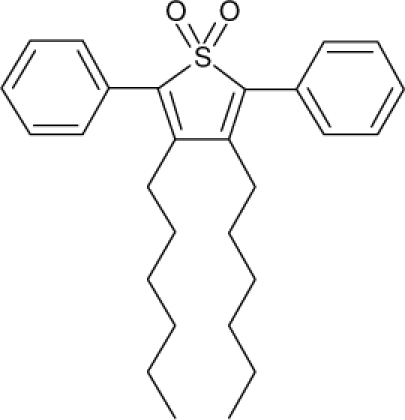					
9:1		495	150	---	SC	49
T5oxPh/PC	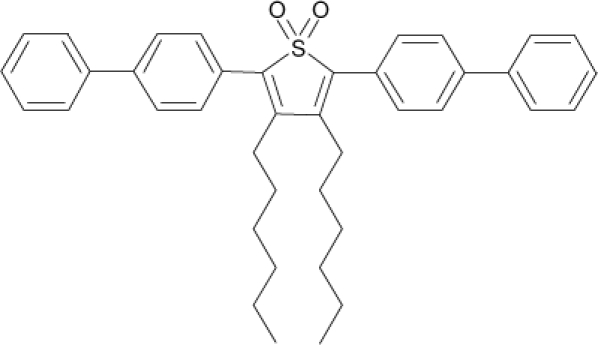					
9:1		545	366	---	SC	49
BP1T	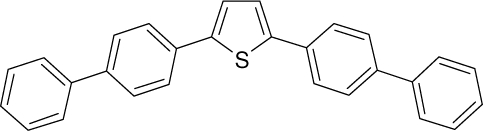	462	13	---	C	50
CCN-DPDSB	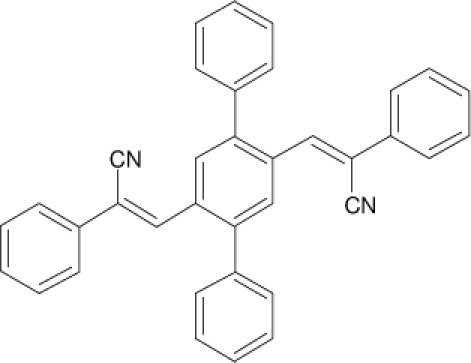	469	39.5		C	51
BPCz	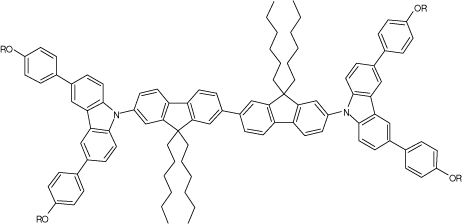	421	4		SC	52

**Material (3.b)**	**Structure^a^**	***λ*ASE^b^**	***I*th-ASE^c^**	***t*1/2-ASE^d^**	**Type of sample^e^**	**Ref^f^**
BUEH-PPV	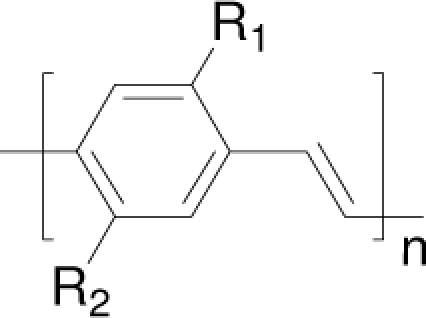	562	0.6	---	SC	53
MEH-PPV	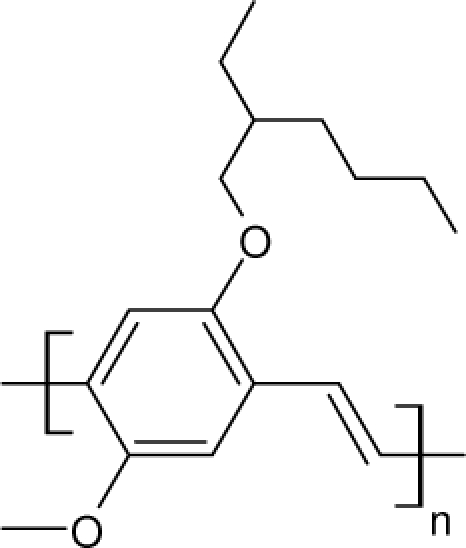	625	4	---	SC	6
PFO	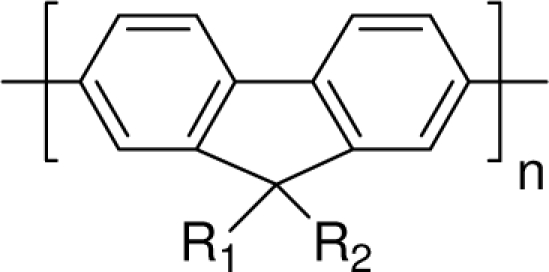	466	2	---	SC	54
F8DP	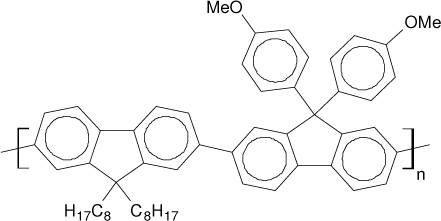	452	0.63	---	SC	55 55
F8BT	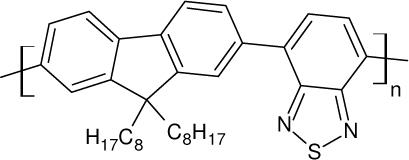	576	2.8	---	SC	56
Copolimer						
Dow Red F		685	2.8	---	SC	56
PHSAF	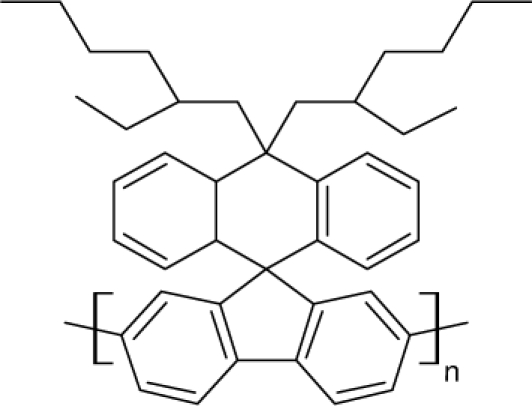	445	13	---	SC	57
TFB	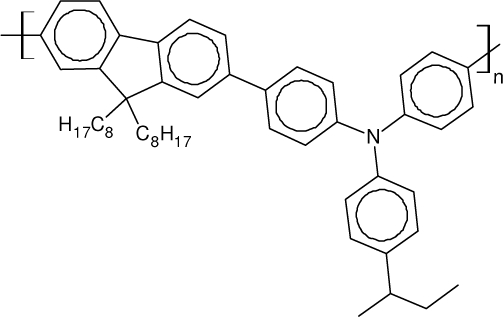	459	250	---	SC	58
PFC	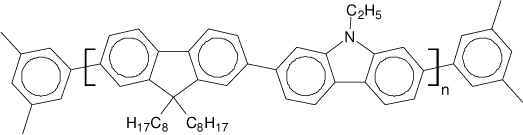	454	60	---	SC	59

a Chemical structure of the active molecule;

b λ_ASE_: ASE Wavelength (nm);

c *I*_th-ASE_: Pump intensity threshold for ASE observation (kW/cm^2^);

d *t*_(1/2)-ASE_: photostability halflife (pump pulses);

e Type of sample: SC (spincoating), C (single crystal);

f Ref: Reference.
